# Immunohistochemical investigation of cell cycle and apoptosis regulators (Survivin, β-Catenin, P53, Caspase 3) in canine appendicular osteosarcoma

**DOI:** 10.1186/1746-6148-8-78

**Published:** 2012-06-11

**Authors:** Laura Bongiovanni, Francesca Mazzocchetti, Daniela Malatesta, Mariarita Romanucci, Andrea Ciccarelli, Paolo Buracco, Raffaella De Maria, Chiara Palmieri, Marina Martano, Emanuela Morello, Lorella Maniscalco, Leonardo Della Salda

**Affiliations:** 1 Department of Comparative Biomedical Sciences, Faculty of Veterinary Medicine, University of Teramo, piazza Aldo Moro, 45, 64100, Teramo, Italy; 2 Department of Communication Sciences, University of Teramo, Campus Coste Sant’Agostino, 64100, Teramo, Italy; 3 Department of Animal Pathology, Faculty of Veterinary Medicine, University of Torino, via L. Da Vinci 48, 10095, Grugliasco, TO, Italy

## Abstract

**Background:**

Osteosarcoma (OSA) represents the most common canine primary bone tumour. Despite several pathways have been investigated so far, few molecules have been identified as prognostic tools or potential therapeutic targets, and there is still the need to find out molecular pathways with specific influence over OSA progression to facilitate earlier prognosis and treatment.

Aims of the present study were to evaluate the immunohistochemical pattern and levels of expression of a panel of molecules (survivin, β-catenin, caspase 3 -inactive and active forms- and p53) involved in cell cycle and apoptosis regulation in canine OSA samples, known to be of interest in the study also of human OSA, and to detect specific relations among them and with histological tumour grade, disease free interval (DFI) and overall survival (OS).

**Results:**

Nuclear β-catenin immunostaining was detected in normal osteoblasts adjacent to the tumour, and in 47% of the cases. Cytoplasmic and/or membranous immunostaining were also observed. Nuclear survivin and p53 positive cells were found in all cases. Moderate/high cytoplasmic β-catenin expression (≥10% positive cells) was significantly associated with the development of metastasis (P = 0.014); moderate/high nuclear p53 expression (≥10% positive cells) was significantly associated with moderate/high histological grade (P = 0.017) and shorter OS (P = 0.049). Moderate/high nuclear survivin expression (≥15% positive cells) showed a tendency toward a longer OS (P = 0,088).

**Conclusions:**

The present results confirmed p53 as negative prognostic marker, while suggested survivin as a potential positive prognostic indicator, rather than indicative of a poor prognosis. The detection of nuclear β-catenin immunostaining in normal osteoblasts and the absent/low expression in most of the OSAs, suggested that this pathway could not play a major role in oncogenic transformation of canine osteoblasts. Further studies are needed to confirm these hypotheses.

## Background

Osteosarcoma (OSA) represents the most common primary bone tumour of both dog [1] and childhood/adolescence [2]. Numerous histological and biological features have been shown to be shared by canine and human OSA to date [3-8], even if further studies are warranted to define more precisely similarities and differences of OSA in the two species, so as to really consider the dog as a valuable spontaneous tumour model for human OSA. Even if several pathways have been investigated in canine OSA so far, few molecules have been identified as prognostic tools or potential therapeutic targets [5,9-18]. Leading on from this, there is still the need to investigate molecular pathways with specific influence over canine OSA progression to facilitate earlier prognosis and treatment.

The goal of the present study was to evaluate a panel of molecules involved in cell cycle and apoptosis regulation in canine OSA samples, known to be of interest in the study also of human OSA. Among these molecules, of particular significance, the role of β-catenin, and the related canonical Wnt pathway, has been largely investigated in human OSA [19-26], but its exact role during neoplastic transformation of osteoblast and OSA progression still remains a debated item. While several studies indicated an important contribution of the activation of the Wnt pathway in OSA development [19-25,27,28], Cai et al. [26] have recently argued the opposite, suggesting that the loss of Wnt pathway activity, which is required for osteoblast differentiation, may contribute to OSA development. Consequently, in contrast to other tumors, β-catenin might not play an oncogenic role in OSA cells [26,29].

Survivin has been recently proposed as a prognostic marker in human [30-35] and canine [36] OSA, but contrasting results have been observed concerning the relation between its subcellular localization and a favourable or poor prognosis. One of the most important aspects of the study of survivin expression in cancer is related to its high cancer specificity and consequent potential role as therapeutic target [37,38]. Survivin has been identified as a target gene of the β-catenin pathway [39], and it acts as a cell cycle regulator and apoptosis inhibitor, even if different isoforms with different functions of the molecule have been described [40].

Loss of p53 tumour suppressor gene functions have been frequently reported in OSA, and mutated p53 has been reported in both human and canine OSA [41]. An interaction between p53 and β-catenin pathway has been proposed to play a key role in osteoblast differentiation and maintenance of bone tissue homeostasis [42]. Furthermore, activation of the Wnt/β-catenin signalling has been shown to cause p53 accumulation [43].

The aims of the present study were to evaluate the immunohistochemical pattern and levels of expression of survivin, β-catenin, caspase 3 (inactive [procaspase 3] and active forms), and p53 in canine OSA samples, to reveal specific relations among them and with histological tumour grade, disease free interval (DFI) and overall survival (OS). The attempt is to contribute to a better understanding of both the pathogenesis of canine OSA and the role of the investigated molecules, in particular establishing their role as prognostic biomarkers and/or potential therapeutic targets. Interestingly, in this study, the expression of nuclear survivin was mainly found in less malignant OSA cases, and β-catenin did not seem to play a major role in oncogenic transformation of canine osteoblasts, while nuclear p53 was confirmed to represent a negative prognostic marker.

## Results

### Histological examination

OSAs were classified according to WHO classification [44], as: 5 productive osteoblastic, 2 non productive osteoblastic, 1 telengectatic, 2 mixed, 3 fibroblastic, 1 undifferentiated, 1 chondroblastic, and 2 giant cell OSA. According to the Kirpensteijn’s grading system [45], 4 cases were classified as grade I, 7 as grade II, and 6 as grade III OSA (Table 1).

**Table 1:** Patients data

	**Breed**	**Sex**	**Age (years)**	**Localization**	**Histological type**	**Post-surgical treatment**	**DFI (days)**	**Overall survival (days)**	**Development of metastasis**
**Grade I**									
n°1	New Found Land	m	11	Left Proximal humerus	fibroblastic osteosarcoma	doxorubicin	1033	1033	No
n°2	Belgian Shepherd Malinois	m	6	Left distal tibia	productive osteoblastic osteosarcoma	cisplatin/doxorubicin	438	449	Yes
n°3	Great Dane	m	5	Left distal tibia	fibroblastic osteosarcoma	cisplatin/doxorubicin	1250	1250	No
n°4	Mixed	m	4	Left distal radius	nonproductive osteoblastic osteosarcoma	cisplatin/doxorubicin	214	1089	Yes
**Grade II**									
n°5	Great Dane	f	4	Right distal radius	chondroblastic osteosarcoma	cisplatin/doxorubicin	89	168	Yes
n°6	Mixed	m	7	Distal ulna	mixed osteosarcoma	cisplatin/doxorubicin	553	553	No
n°7	Mixed	f	8	Left Proximal humerus	mixed osteosarcoma	doxorubicin	503	503	No
n°8	Rottweiler	f	7	Right proximal femur	poorly differentiated osteosarcoma	doxorubicin	623	715	Yes
n°9	German Sheperd	m	8	Right proximal humerus	fibroblastic osteosarcoma	cisplatin/doxorubicin	496	496	No
n°10	Maremma Sheepdog	m	9	Right distal radius	teleangectatic osteosarcoma	cisplatin/doxorubicin	261	261	No
n°11	Rottweiler	f	7,5	Right distal radius	productive osteoblastic osteosarcoma	cisplatin/doxorubicin	391	391	No
**Grade III**									
n°12	Boxer	f	8	Right distal radius	productive osteoblastic osteosarcoma	cisplatin/doxorubicin	103	169	Yes
n°13	Great Dane	m	10	Left distal radius	productive osteoblastic osteosarcoma	cisplatin/doxorubicin	210	242	Yes
n°14	German Sheperd	f	7	Left distal femur	nonproductive osteoblastic osteosarcoma	doxorubicin	309	335	Yes
n°15	Rottweiler	m	2,5	Distal radius	giant cell type osteosarcoma	cisplatin	75/167	211	Yes
n°16	New Found Retriever	m	7	Left Distal radius	productive osteoblastic osteosarcoma	cisplatin/doxorubicin	126	200	Yes
n°17	Saint Bernard	m	7	Left Distal radius	giant cell type osteosarcoma	cisplatin/doxorubicin	203	203	No

Tumour grade, breed, sex, age, localization, histological type, post-surgical treatment, DFI, overall survival and development of metastasis of the cases included in the present study.

### -catenin expression in canine OSA

**Figure 1:**
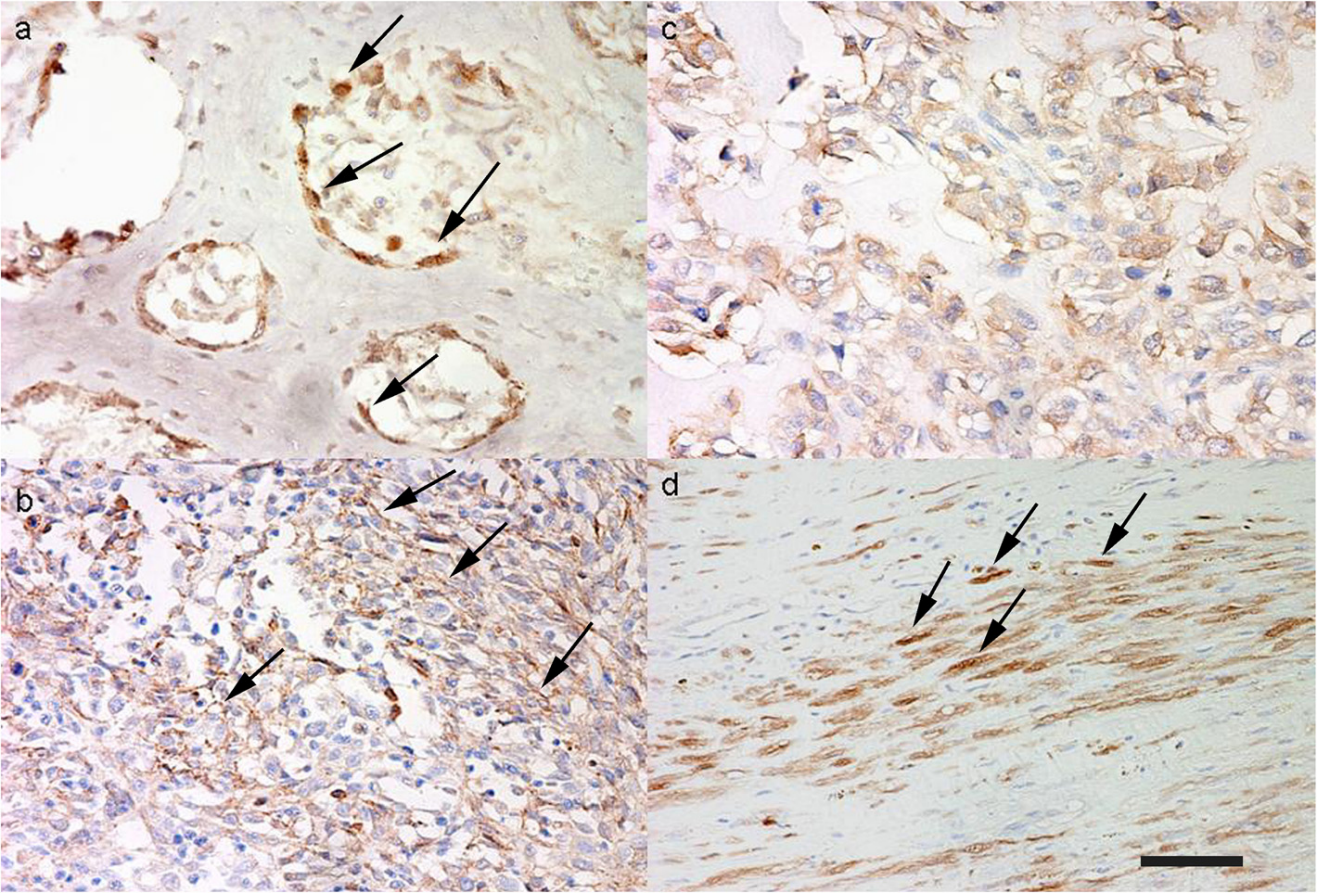
**Immunohistochemical β-catenin expression. a **– Osteoblasts lining bone trabeculae surrounding neoplastic tissue. Intense nuclear immunostaining (arrows) (Bar. 40 μm) **b** – Non-productive osteoblastic OSA. Multifocal and inconsistent membranous immunostaining (arrows) (Bar. 80 μm). **c **- Productive osteoblastic OSA. Diffuse cytoplasmic immunostaining (Bar. 40 μm). **d **– Fibroblastic OSA. Multifocal nuclear immunostaining (arrows) (Bar. 80 μm).

**Figure 2:**
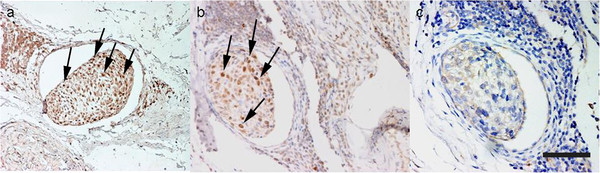
**Immunohistochemistry in neoplastic emboli. a **– Numerous p53-positive nuclei (arrows) (Bar. 160 μm). **b **– Weak cytoplasmic and multifocal nuclear (arrows) survivin immunostaining (Bar. 80 μm). **c **– Weak β-catenin cytoplasmic and no nuclear and membranous immunostaining (Bar. 80 μm).

In normal osteoblasts surrounding bone trabeculae adjacent to the tumours, β-catenin showed an intense nuclear expression, and a less intense cytoplasmic-membranous expression (Figure 1,a). In tumour cells, membranous β-catenin immunostaining was observed in 65% of the cases, inconsistent and multifocal (Figure 1,b), observed in 2/4 grade I, 5/7 grade II and 4/6 grade III cases. Cytoplasmic staining was found in all the samples examinated (Figure 1, c). Nuclear localization was found in only 47% of the cases, observed in 2/4 grade I, 5/7 grade II and 1/6 grade III cases. The number of positive nuclei were very low: 50% grade I showed no positive nuclei and 50% a number of positive nuclei ranging from 0 to 10%; >0 and <10% positive nuclei were found in 5/7 of grade II; while 5/6 grade III did not show any positive nuclei (Tables 2 and 3). Most of the cases showing positive nuclei were fibroblastic OSA (Figure 1, d) (3 cases) or positive nuclei were found in spindle shaped cells present in the case of undifferentiated OSA, and in two cases of productive OSA. Positive nuclei were not found among giant cells, nor among cells of neoplastic emboli, where only cytoplasmic immunostaining was observed (Figure 2, a).

**Table 2:** Immunohistochemical results 1

		**β-Catenin**			**P53**	**Procaspase 3**	**Active Caspase 3**	**Survivin**	
**GRADE**	**N°**	**Nuclear**	**Membranous**	**Cytoplasmic**				**Cytoplasmic**	**Nuclear**
I	4	2 (50%)	2 (50%)	4 (100%)	2 (50%)	4 (100%)	4 (100%)	4 (100%)	4 (100%)
II	7	5 (71%)	5 (71%)	7 (100%)	6 (86%)	7 (100%)	7 (100%)	7 (100%)	7 (100%)
III	6	1 (17%)	4 (67%)	6 (100%)	6 (100%)	6 (100%)	6 (100%)	6 (100%)	6 (100%)

Positive samples for each molecules investigated grouped in different grades, according to Kirpensteijn’s grading system [45].

**Table 3:** Immunohistochemical results 2

**Grade**	**β-catenin**			**p53**
	**N**	**M**	**C**	
**I**	-	-	-	-
0	2 (50%)	2 (50%)	-	2 (50%)
>0 - <10%	2 (50%)	1 (25%)	1 (25%)	2 (50%)
≥10 - <25%	-	-	-	-
≥25 - <50%	-	1 (25%)	1 (25%)	-
≥50%	-	-	2 (50%)	-
**II**				
0	2 (28.6%)	1 (14.3%)	-	1 (14.3%)
>0 - <10%	3 (42.8%)	1 (14.3%)	2 (28.6%)	1 (14.3%)
≥10 - <25%	1 (14.3%)	1 (14.3%)	2 (28.6%)	3 (42.8%)
≥25 - <50%	1 (14.3%)	1 (14.3%)	1 (14.3%)	2 (28.6%)
≥50%	-	2 (28.6%)	2 (28.6%)	-
**III**				
0	5 (83.3%)	2 (33.3%)	-	-
>0 e <10%	-	2 (33.3%)	1 (16.7%)	-
≥10 - <25%	1 (16.7%)	1 (16.7%)	2 (33.3%)	1 (16.7%)
≥25 - <50%	-	1 (16.7%)	2 (33.3%)	4 (66.6%)
≥50%	-	-	1 (16.7%)	1 (16.7%)

Number of positive cases (and percentage) for each ranges of positive cells observed, grouped in different grades, according to Kirpensteijn’s grading system [45]. N: nuclear; M: membranous; C: cytoplasmic.

By Fisher’s exact test moderate/high cytoplasmic β-catenin expression (≥10% positive cells) in the primary tumours was predictive of development of metastasis (P = 0.014). No relations were found between the semi-quantitative evaluation of β-catenin and the OS by Kaplan-Meier survival analysis.

### Survivin expression in canine OSA

**Figure 3:**
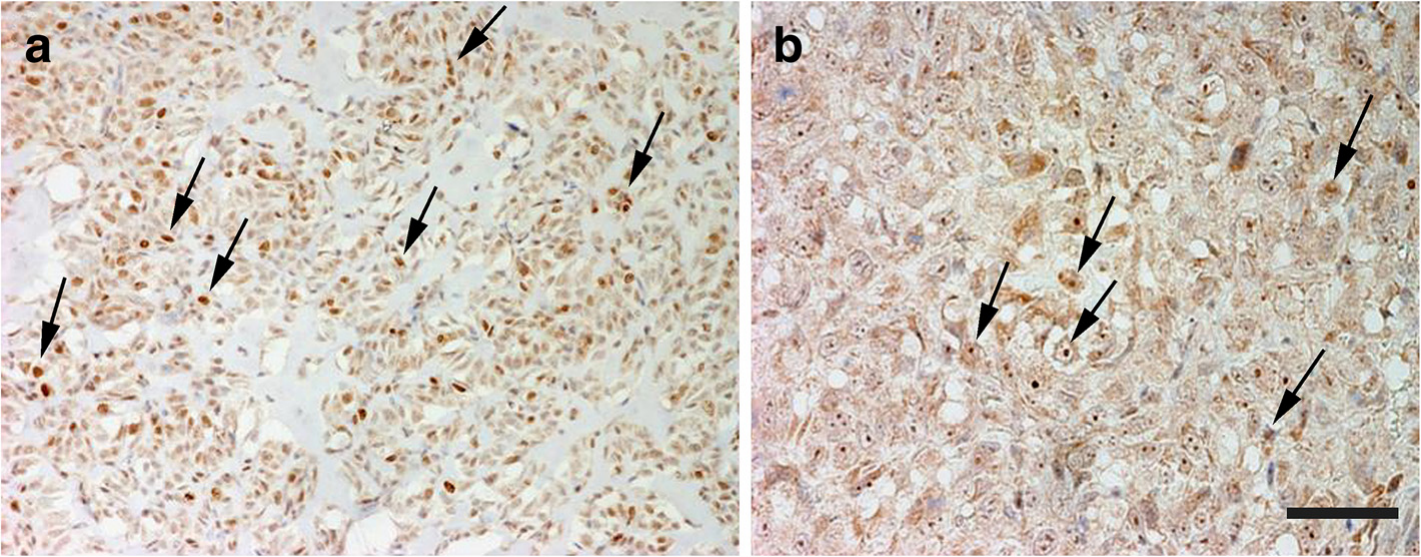
**Survivin and p53 immunohistochemical expression. a **– Productive osteoblastic OSA. Multifocal survivin nuclear immunostaining(arrows) (Bar. 80 μm). **b **– Productive osteoblastic OSA. Numerous p53 positive nuclei (arrows) (Bar. 40 μm).

No staining was found in normal osteoblasts surrounding bone trabeculae adjacent to the tumours, while all samples investigated showed both nuclear and cytoplasmic survivin immunostaining. Cytoplasmic survivin expression was very high (≥75% positive cells) in all the cases evaluated. Nuclear staining was more intense than cytoplasmic, with positive mitotic figures observed among all the tumours (Figure 3, a). Positive nuclei were also multifocally and inconsistently found in the giant cells. Numerous positive nuclei were present among cells of neoplastic emboli (Figure 2, b).

**Figure 4:**
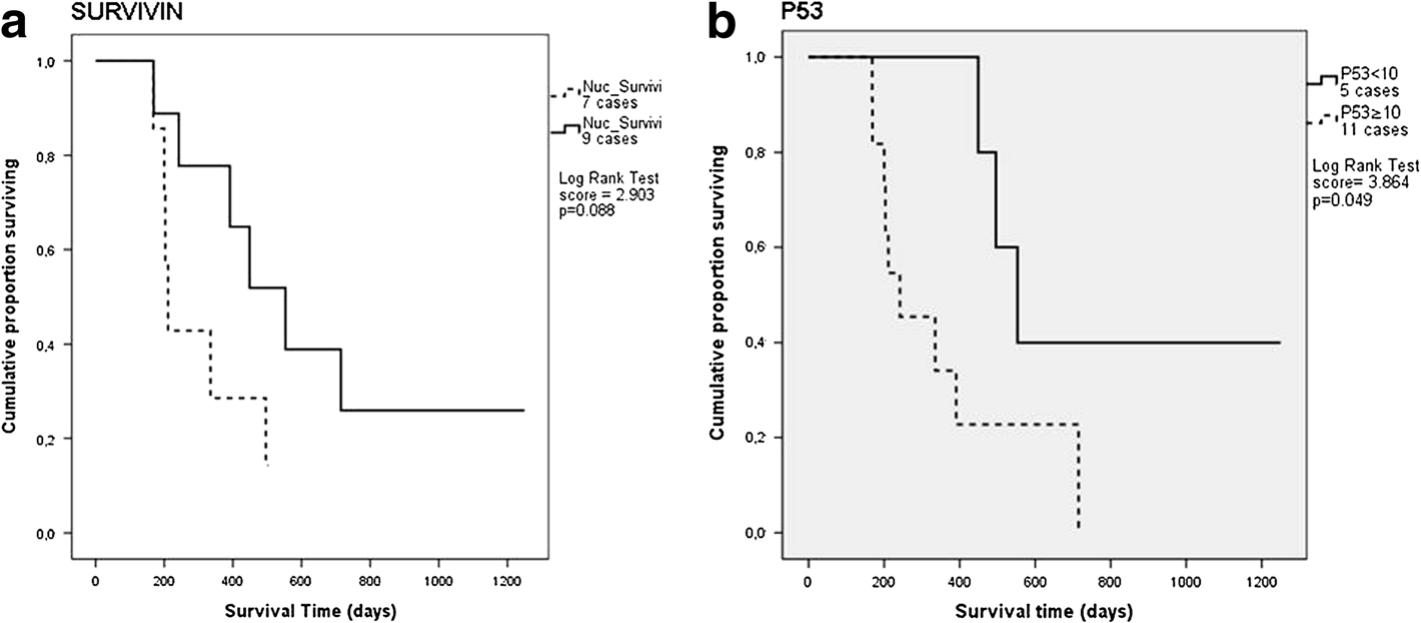
**Kaplan-Meier plots showing influence on survival of survivin and p53. (a) **Nuclear survivin expression score ≥15% showed a tendency toward a longer survival time (P = 0,088). **(b) **P53 nuclear expression score ≥10% appeared to be significantly associated to a longer post-surgical OS (P = 0.049).

Survivin nuclear expression ranged from 55.55% to 3.86%, with a mean value within the three different groups of 38.22% ± 17.5 (grade I), 16.56% ± 7.9 (grade II) and 13.59% ± 9 (grade III) (mean value ± standard deviation). Statistically significant correlation, using Pearson’s correlation coefficient, was observed between nuclear survivin expression and active caspase 3 expression (ρ = 0.696; P = 0.008), longer DFI (ρ = 0.741; P = 0.001) and OS (ρ = 0.748; P = 0.001); the relation between nuclear survivin expression and active caspase 3 expression seems to be confirmed by the Kendall’s Tau_b_ non parametric test (τ_b_ = 0.513; P = 0.015), while the same test gives less significant results with longer DFI (P = 0.177) and OS (P = 0.150). The Kaplan-Meier analysis and Log-Rank test (P = 0.088) (Figure 4, a) showed a tendency toward a longer OS in subject with moderate/high nuclear survivin expression (≥15% positive cells).

### p53 expression in canine OSA

Positive nuclei were observed in 82% of cases, found in 2/4 grade I, 6/7 grade II and 6/6 grade III cases (Tables 2 and 3). Most of giant cells were negative, and scattered positive nucleoli were also observed (Figure 3, b). Numerous cells within neoplastic emboli showed positive nuclei (Figure 2, c).

A high nuclear p53 expression (≥25% positive cells) was significantly associated with a high histological grade (III) (P = 0.035), while moderate/high nuclear expression (≥10% positive cells) was significantly associated with a moderate/high histological grade (II and III) (P = 0.017) and longer OS (P = 0.004). The Kaplan-Meier analysis and Log-Rank test (P = 0.049) also showed longer OS in subject having moderate/high nuclear p53 expression (≥10% positive cells) (Figure 4, b).

### Caspase 3 expression in canine OSA

A diffuse procaspase 3 cytoplasmic immunostaining was found in all the cases investigated; while multifocal active caspase 3-positive nuclei were found in all the neoplastic tissues, but none in normal osteoblasts surrounding bone trabeculae adjacent to the tumours.

Active caspase 3 expression ranged from 65.41% to 5.48%, with a mean value within the three different groups of 53.82% ± 16.4 (grade I), 30.41% ± 23.4 (grade II) and 13.78% ± 14.4 (grade III) (mean value ± standard deviation).

No relations were found between the semi-quantitative evaluation of procaspase 3 and active caspase 3 and the investigated clinico-pathological parameters.

## Discussion

The present study demonstrated and confirmed the expression of several important molecules in canine OSA, showing a potential prognostic value of some of them, that could also be considered as possible candidates for a therapeutic target.

Even though the importance of the Wnt/β-catenin pathway in bone development and homeostasis is well defined [46,47], the role of β-catenin in the development and progression of bone tumours is not completely known [48]. Since OSA formation is probably the result of an altered bone regulation, it seems likely that deregulation of the Wnt signalling could be associated with the pathogenesis of OSA. The presence of intense β-catenin-positive nuclei in normal osteoblasts observed in the present study would confirm the fundamental role of the Wnt/β-catenin pathway in the maintenance of these cells. Indeed, nuclear β-catenin expression has been considered a hallmark of activation of the Wnt/β-catenin pathway [26,49] or mutations of β-catenin or molecules of its degradation complex [50]. Rare or completely absent positive nuclei were observed in the most malignant cases examinated herein, even if no significant association with the OS was observed, as well as in neoplastic emboli, suggesting that neither mutations nor activation of the Wnt/β-catenin pathway would occur in these cells. In accordance with that, no mutation has been recently identified in canine β-catenin exon 3, similarly to human OSA [19], even if a limited number of cases were examinated, and a mainly cytoplasmic, but rare nuclear localization of the protein was detected [51]. Furthermore, no differences in nuclear β-catenin immunohistochemical expression were observed in samples obtained from two populations of canine OSA patients with normal or high level of serum alkaline phosphatase concentration [52], known to have negative prognostic value in canine osteosarcoma patients [53]. These results seem to be in agreement with recent investigations in human OSA [26], where no β-catenin nuclear expression was observed in 90% of human high grade OSA biopsies examinated and inhibition of the Wnt/β-catenin pathway has been demonstrated to be important in osteoblast neoplastic transformation [26]. These observations seemed to confirm the hypothesis that inactivation of the Wnt/β-catenin pathway could be essential in OSA progression, differently to what is usually observed in other cancers [54], opening to new potential therapeutic strategies. Specific small molecule compounds that activate Wnt/β-catenin signaling in a highly cell-type specific manner (human OSA U2OS cell line) have recently been identified [55], that could induce the activation of the Wnt/β-catenin pathway and provide new therapeutic opportunities. Interestingly, high β-catenin cytoplasmic expression rate was significantly related to the development of metastasis. High levels of cytoplasmic protein, in absence of nuclear staining, could be related to a reduced membranous β-catenin, reported to be associated to invasion and metastatic potential in human OSA [21], however, an inhibition of β-catenin nuclear translocation may also be hypothesized.

Similarly to what has previously been observed in human [30,31] and canine [36] OSA, both cytoplasmic and nuclear survivin expression pattern was observed in canine OSA. Mechanisms controlling survivin nuclear and/or cytoplasmic localization in tumour cells are often a debated question, since the item could acquire a different prognostic significance depending on the tumour type [56]. Our results seem to be in contrast with a recent study in which a correlation between survivin immunohistochemical expression and histological grade and mitotic index has been reported in canine OSA tissue samples [36]. Similarly, divergent results have been published concerning the relation between survivin expression and malignancy of human OSA [30,32-34]. Disagreements could be related to the presence of several survivin isoforms, with different, even opposite, functions, and this might indicate different activities of survivin within the cell. In particular, two survivin variants potentially act in an opposite way to the other three variants, with a potential proapoptotic function: Survivin-2α may attenuate the anti-apoptotic activity of full length survivin [57], while survivin-deltaEx3 has been shown to have apoptotic functions [58]. Noteworthy, the polyclonal antibody used in the present study recognizes all survivin splice variants, and decreased nuclear survivin expression score was found among more malignant grade III cases of canine OSA, as well as higher nuclear survivin expression appeared to be related to a longer post-treatment OS, similarly to that observed by Trieb et al. [30] in the human counterpart. A significant association was revealed between nuclear survivin expression and the activation of caspase 3, most probably indicative of apoptotic pathway activation, further supporting the hypothesis that nuclear survivin expression could be considered a positive prognostic marker in canine OSA. Indeed, despite a diffuse cytoplasmic staining of its inactive form (procaspase 3) observed in all the cases examinated, a progressive decreasing of the active caspase 3 mean values was observed in OSA from grade I to III.

Differently to primary tumours, a frequent nuclear survivin immunostaining was observed among neoplastic emboli, suggesting a different behaviour of the protein in OSA metastatic spread. Therefore, further investigations on survivin expression in metastatic OSA are warranted, in order to understand its functions and role in the response to postoperative chemotherapy, since adjuvant treatment is mainly aimed at controlling the metastatic disease, and survivin has been frequently associated to resistance to chemotherapy [59,60]. Recently, this has been demonstrated also in canine OSA cell lines (Abrams and D17), where after survivin inhibition an increase of chemosensitivity to both doxorubicin and carboplatin has been observed [36]. Survivin has been proposed as a valuable target for anticancer therapy [37], and several molecules with a direct or indirect effect on survivin expression have been identified to date, some of which have been tested in clinical trials with encouraging results [61]. Decreasing survivin expression through STAT3 [5,62] or Hsp90 [63] inhibition in canine OSA cell lines has been recently proposed as a possible new therapeutic approach.

There is a strong evidence of the involvement of p53 mutations in the development of canine OSA [41,64,65], as well as in the human counterpart [66,67]. In the present study, highest levels of expression of nuclear p53 were significantly associated with a shorter post surgical OS, and appeared to be related to the development of metastasis, confirming the importance of p53 as a negative prognostic marker in canine OSA, in accordance to previous published data regarding canine OSA, suggesting that, similarly to human OSA [68,69], alterations in p53 functions are associated with highly aggressive tumour behaviour [70,71]. Deregulation of the p53-survivin subsystem has been proposed to play an important role in several tumour types [72], since, WTp53 is able to repress survivin expression at both the mRNA and protein level [40]. In spite of this, our results suggests that different mechanisms could regulate survivin expression in canine OSA.

## Conclusions

Data obtained from the present immunohistochemical study suggest survivin as a potential positive prognostic marker, rather than a marker of poor prognosis, even if its presence in metastatic emboli suggests its possible involvement in the malignant progression of canine OSA. Further investigations, in particular on the expression and roles of its different isoforms, should be performed in order to clarify if survivin could represent a valid biomarker in this kind of tumour.

Lastly, the present data suggest that β-catenin, a key molecule for normal osteoblast differentiation, could not play a key role in the neoplastic transformation of canine osteoblasts, as recently suggested in human OSA. Further studies should be done in order to confirm this hypothesis.

## Methods

### Tissue Samples and Clinical data

This retrospective study looked at of 17 canine OSA surgical samples, provided by the Department of Animal Pathology, Faculty of Veterinary Medicine, University of Torino (Italy), for which survival data were also provided. All the dogs included in the present study had an appendicular OSA (histologically confirmed) at presentation and no evidence of metastasis. Clinical staging included history, physical exam, complete blood count, serum biochemical profile, urinalysis and abdominal ultrasound. Limb (latero-lateral [LL] and antero-posterior [AP] views) and chest (right and left LL, and dorso-ventral [DV] views) radiographic evaluation was performed to examine features and extension of the tumour and presence of lung metastasis, respectively. Computed Tomography was performed in case of radiographic suspicion of lung metastasis. Regional lymph nodes were aspirated and cytologically examined when enlarged at clinical palpation. Initial diagnosis of the tumour was attempted by fine needle aspiration and cytology but, for a more specific identification of the tumour type, a preoperative biopsy was obtained in all cases using a Jamshidi needle and submitted to histopathology. Histopathology was performed also on the entire tumour specimen (in case of limb amputation or limb sparing using an allograft to substitute the tumour segment) or on a sample of the tumour (in case of limb sparing using the pasteurized neoplastic autograft), in order to confirm the diagnosis [73,74]. All dogs included in the present study were surgically treated (amputation or limb sparing) before receiving adjuvant chemotherapy using doxorubicin (30 mg/m2, 4–5 administrations, 21 days apart) or cisplatin (70 mg/m2, 4–5 administrations, 21 days apart) as a single agent, or a combination of cisplatin and doxorubicin (4 cycles, 21 days apart, each cycle consisting of cisplatin 50 mg/m2 at day 1 and doxorubicin 15 mg/m2 at day 2). One of the three chemotherapy protocols was chosen based on the dog clinical status and owners compliance, since no protocol has been demonstrated to be superior [75-78]. Besides, histopathological tumour grade never influenced the choice of the chemotherapeutic protocol adopted. Canine patients were clinically and radiographically examined every 3 months during the first year after the conclusion of chemotherapy and then every 6 months for a minimum of 2 years. DFI (disease free interval) was considered as the period in days from surgery to recurrence and/or metastasis development, while OS (overall survival) as the period in days from surgery to death for tumour-related causes; for dogs still alive at the time of writing, OS was the number of days from surgery to the last clinical examination (patients’ data are summarized in Table 1).

### Histological examination

All specimens were routinely fixed in 10% formalin, embedded in paraffin wax, and 4–5 μm-thick sections were examined using haematoxylin and eosin (H&E) staining and visualized by light microscopy. Tumours were histologically classified according to the World Health Organization (WHO) criteria [44], and histological grade was determined according to the system proposed by Kirpensteijn et al. [45].

### Immunohistochemical Examination

**Figure 5:**
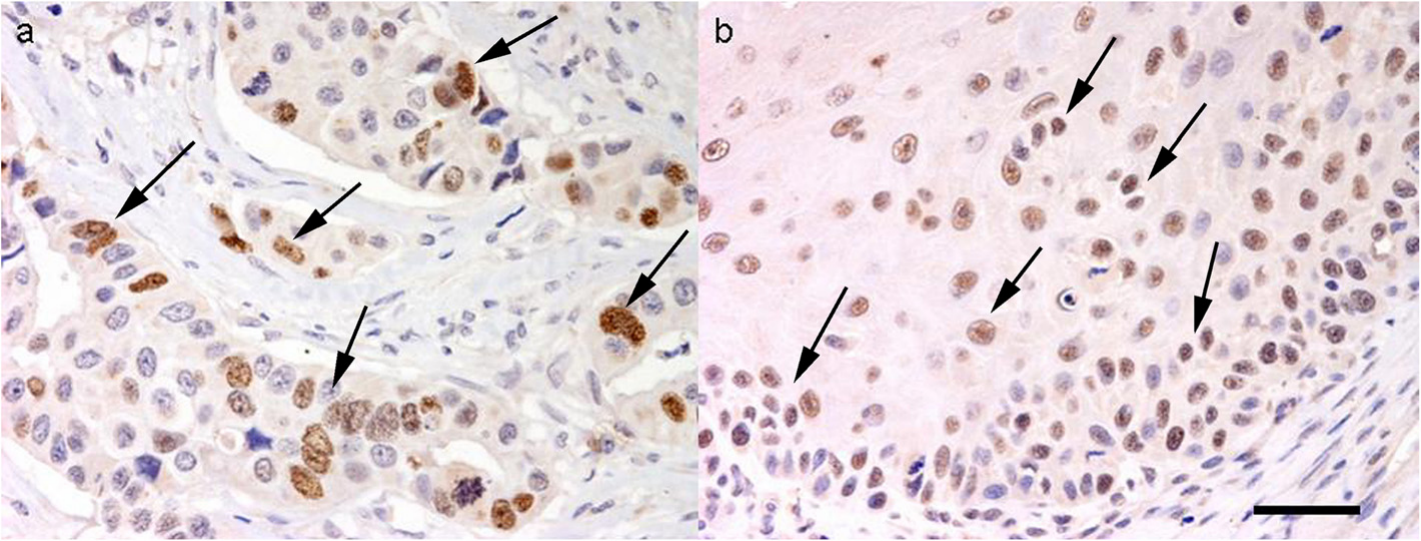
**Positive control tissues for p53. a**- Human mammary carcinoma. p53-positive nuclei (arrows) (Bar. 40 μm). **b **– Canine nail bed squamous cell carcinoma. Numerous p53-positive nuclei (arrows) (Bar. 40 μm).

Dewaxed and rehydrated tissue sections were immunostained by the streptavidin-biotin peroxidase complex (SAB) method, using specific primary antibodies (Abs) reported in Table 4, incubated overnight in a humidified chamber at 4°C. Immunohistochemical analysis followed procedure as reported in previous studies [79]. Endogenous peroxidase was blocked with H_2_O_2_ 3% in absolute methanol for 45 min. Antigen retrieval was undertaken by heat-treating sections in citrate buffer at pH 6 (β-catenin, caspase 3) or Tris-EDTA pH 9.0 (p53) in a microwave oven for 5min. (3 cycles) or in citrate buffer at pH 6 in a pressure cooker for 20 min. (survivin). Presence of antibody binding was visualized with 3-3’-diaminobenzidine (DAB, D5905, Sigma-Aldrich, St. Louis, MO, USA) solution, which was applied for 5 min, followed by a light counterstain with Mayer’s haematoxylin (Merck, Darmstadt, Germany) for 1 min. Positive control slides were used for each Abs using a tissue of known pattern of expression of each molecules (Table 4). Anti-p53 antibody validation was performed using samples of human mammary carcinoma (Figure 5, a) and a samples of canine nail bed squamous cell carcinoma (SCC) (Figure 5, b) then used as positive control. A negative control was performed in all instances by omitting the primary antibody and incubating tissue sections with Tris Buffered Saline (TBS); for active caspase 3, an irrelevant antibody directed against an unrelated antigen (mouse anti human desmin monoclonal antibody – Dako, Glostrub, Denmark) was also used.

**Table 4:** Primary antibodies used in immunohistochemical analysis

**MOLECULE**	**TYPE OF Ab**	**COMPANY**	**WORKING DILUTION**	**POSITIVE CONTROL**
β-catenin	Rabbit PAb	Santa Cruz Biotecnology	1:250	Normal canine skin [80]
Survivin	Rabbit PAb	NOVUS Biologicals	0.7 μg/ml	Canine cutaneous SCC [79]
p53	Rabbit PAb	NovoCastra	1:400	Canine nail bed SCC (Figure 5)
Procaspase 3	Rat MAb	NovoCastra	1:85	Normal canine tonsil [81]
Active Caspase 3	Mouse MAb	Oncogene	1:200	Canine breast carcinoma [81]

Clonality, company, working dilutions and positive controls used for each antibody. PAb: polyclonal antibody; MAb: monoclonal antibody. SCC: squamous cell carcinoma.

### Quantification of Immunostaining and Statistical Analysis

Neoplastic tissues were scored by two pathologists as follow.

Survivin and caspase 3 expression was evaluated by counting the number of positive nuclei in 10 HPF at 400X, counting approximately 1000 cells, and expressed as a percentage. Cytoplasmic survivin expression was diffusely present in 100% of the cases evaluated.

For p53 only nuclear staining was considered as positive, since nuclear immunostaining is suggestive of its mutation [68]; β-catenin expression was classified as membranous (localised at cell-cell boundaries), cytoplasmic (uniformly distributed throughout the cytoplasm) and nuclear. A semi-quantitative immunohistochemical assessment was performed and samples were subdivided based on the protein expression levels in 4 ranges for β-catenin and p53 (absent: no positive cells; low: >0% - <10% positive cells; moderate: ≥10% - <50% positive cells; high: ≥50% positive cells) and 5 ranges for procaspase 3 and survivin (absent: no positive cells; low: >0% - <10% positive cells; moderate: ≥10% - <50% positive cells; high: ≥50% - <75% positive cells; very high ≥75% positive cells).

Fisher's exact test was applied to evaluate the association between the expression levels of the investigated molecules and clinico-pathological parameters. Pearson correlation test and Kendall’s Tau_b_ test were applied to examine the relations between the expression levels of nuclear survivin and p53, DFI, OS. Kaplan Meier analysis was used to estimate survival, and the significances of the differences were determined by the Log-Rank test. For this purpose, the cases were grouped according to the expression score as follows: <10% positive cells (absent + low semiquantitative evaluation) versus ≥10% positive cells (moderate + high semiquantitative evaluation) for p53 and β-catenin; <15% positive cells (absent + low semiquantitative evaluation) versus ≥15% positive cells (moderate + high semiquantitative evaluation) for survivin; or <25% positive cells (absent/low + moderate semiquantitative evaluation) versus ≥25% positive cells (high semiquantitative evaluation). Analyses were performed using the IBM SPSS19 statistical software, and the conventional 5% level was used to define statistical significance.

## Authors’ contributions

LB participated in the design of the study and drafted the manuscript. FM carried out the immunohistochemistry and helped to draft the manuscript. MD participated in the design of the study. RM participated in the design of the study and helped to draft the manuscript. AC performed the statistical analysis and participate in the interpretation of the results. PB performed surgical treatment and adjuvant chemotherapy, providing follow-up and clinical data of the patients. RDM provided technical support. CP provided technical support. MM performed surgical treatment and adjuvant chemotherapy, providing follow-up and clinical data of the patients. ME performed surgical treatment and adjuvant chemotherapy, providing follow-up and clinical data of the patients. ML provided technical support. LDS conceived of the study, participated in its design and coordination and helped to draft the manuscript. All authors read and approved the final manuscript.
